# Applications of Lipidomics to Age-Related Musculoskeletal Disorders

**DOI:** 10.1007/s11914-021-00656-0

**Published:** 2021-02-16

**Authors:** Chenglin Mo, Yating Du, Thomas M. O’Connell

**Affiliations:** 1 Bone-Muscle Research Center, College of Nursing and Health Innovation, University of Texas at Arlington, Arlington, TX USA; 2 Department of Otolaryngology, Head & Neck Surgery, Indiana University School of Medicine, Indianapolis, IN USA

**Keywords:** Osteoporosis, Sarcopenia, Biomarker, Lipidomics and metabolomics

## Abstract

**Purpose of Review:**

The goal of this review is to highlight the need for new biomarkers for the diagnosis and treatment of musculoskeletal disorders, especially osteoporosis and sarcopenia. These conditions are characterized by loss of bone and muscle mass, respectively, leading to functional deterioration and the development of disabilities. Advances in high-resolution lipidomics platforms are being used to help identify new lipid biomarkers for these diseases.

**Recent Findings:**

It is now well established that bone and muscle have important endocrine functions, including the release of bioactive factors in response to mechanical and biochemical stimuli. Bioactive lipids are a prominent set of these factors and some of these lipids are directly related to the mass and function of bone and muscle. Recent lipidomics studies have shown significant dysregulation of lipids in aged muscle and bone, including alterations in diacylglycerols and ceramides. Studies have shown that alterations in some types of plasma lipids are associated with aging including reduced bone mineral density and the occurrence of osteoporosis.

**Summary:**

Musculoskeletal disorders are a major burden in our society, especially for older adults. The development and application of new lipidomics methods is making significant advances in identifying new biomarkers for these diseases. These studies will not only lead to improved detection, but new mechanistic insights that could lead to new therapeutic targets and interventions.

## Introduction

Musculoskeletal disorders (MSDs) affecting joint, spine, bone, and muscle are a leading cause of disability globally [1]. Although MSDs are prevalent across different ages, the proportion of persons living with MSDs increases with age. For people age 65 and over, approximately three out of four are suffering from MSDs. In addition, the cost of MSDs has become a huge burden for our society and healthcare system. In 2017, the annual total cost of MSDs in the USA was estimated to be around $874 billion, nearly 5.73% of gross domestic product [1].

Among MSDs, osteoporosis and sarcopenia are the conditions most closely associated with aging. Osteoporosis is characterized by low bone mass and deteriorated bone microstructure with an increased susceptibility to fracture. It is estimated that the number of people with low bone mass, a potential precursor to osteoporosis, will grow from 43.4 million in 2010 to 57.8 million in 2030 [1]. Over the same time frame, the number of people with osteoporosis is expected to increase from an estimated 10.2 million in 2010 to 13.6 million in 2030. Osteoporosis leads to a significant increase in morbidity and mortality, with fractures being a common sequela [1]. Fractures most commonly occur in the hip, spine, and forearm [1]. Approximately 50% of women and 25% of men over the age of 50 will suffer from a fracture due to fragile bones.

A common comorbidity of osteoporosis is the loss of muscle mass and muscle strength during aging known as sarcopenia. A recently released report analyzed the prevalence of sarcopenia in the USA using data generated from the National Health and Nutrition Examination Survey (NHANES). The analysis included data from 4011 subjects during the period from 1999 to 2004. The analysis showed that 15.1% of individuals had sarcopenia and those in that group had about twice the odds of hospitalization compared with individuals without sarcopenia [2].

Osteoporosis often develops “silently” with patients often not learning of their condition until a fracture occurs. Similarly, age-related loss of muscle mass also follows a very slow and silent progression. Lean muscle mass starts to gradually decrease around age 30 and follows a continued decline of about 3 to 8% per decade. After age 60, this process may reach 10% per decade [3].

Routine measurements of the changes in bone and muscle mass are important for early diagnosis of osteoporosis and sarcopenia. Osteoporosis is commonly diagnosed by measuring the bone mass density (BMD) using dual-energy X-ray absorptiometry (DEXA). This same method is also applied to the measurement of lean muscle mass. However, there are some limitations of DEXA. Many patients with osteoporosis/sarcopenia have limited access to healthcare services with this expensive technology. This method cannot detect microarchitectural changes in the bone structure, which are an early development of osteoporosis [4]. In sarcopenia, accumulating data shows that it is principally the decreased contractile force, not the loss of muscle size per se, that contributes to disability and to the high healthcare costs in aging populations [5]. Given these limitations with DEXA, it is clear that new diagnostic tools are needed to detect the structural and functional changes of bone and muscle associated with osteoporosis and sarcopenia. Ideally, these diagnostic tools should be accessible, non-invasive, and inexpensive. As muscle and bone are highly metabolic organs, it is sensible to focus on metabolism in the search for diagnostic biomarkers. In particular, the bioactive lipids are an important category of metabolites and recent studies have shown that these lipids may play a significant role in the development and progression of age-related MSDs. The focus of this review is to present the state of the art in the development of novel lipid biomarkers for the detection and diagnosis of osteoporosis and sarcopenia.

## Current Development of Osteoporosis/Sarcopenia Biomarkers

### Lipids as Potential New Biomarkers of Osteoporosis/Sarcopenia

**Fig. 1:**
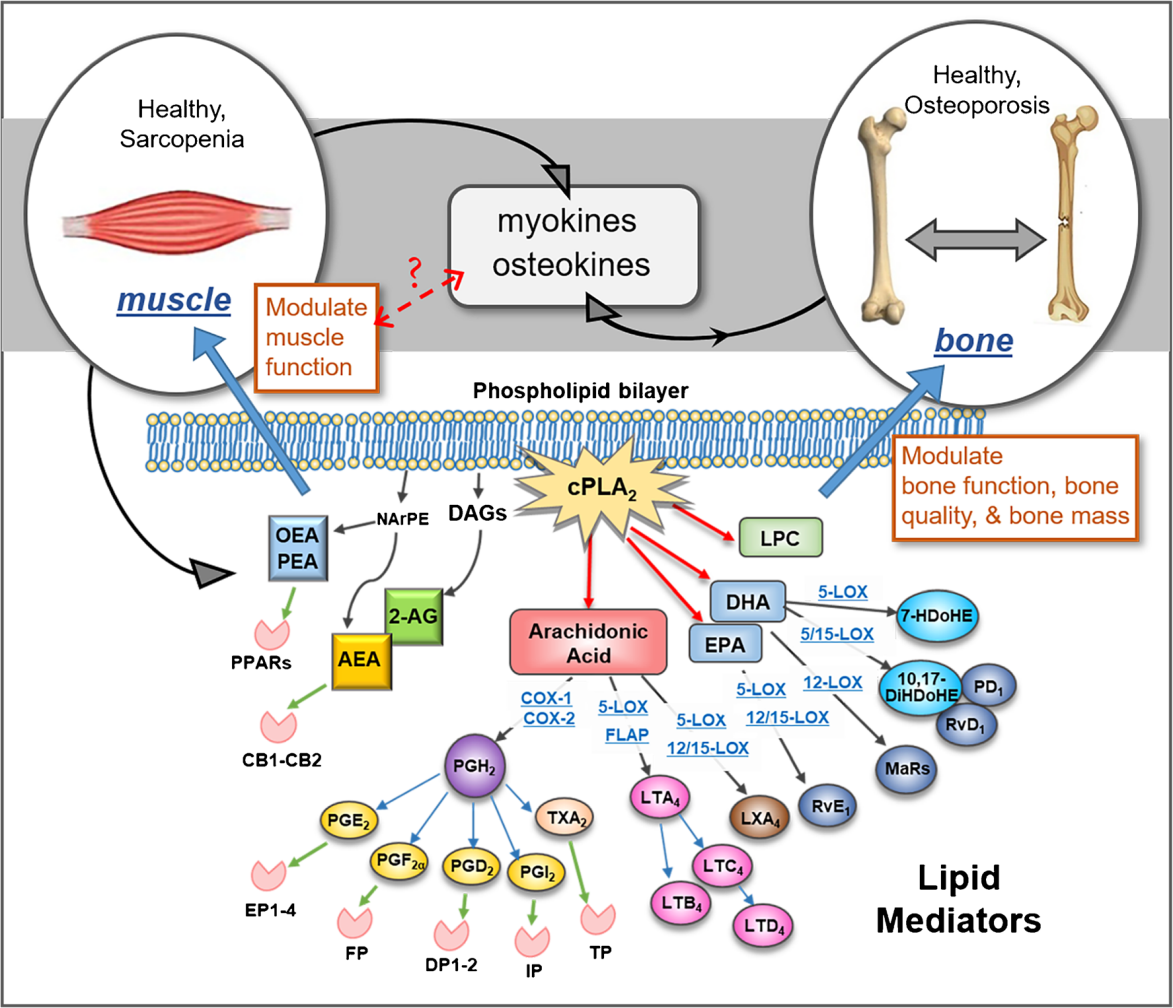
Generation and signaling pathways of some key lipid mediators in bone, muscle, and bone-muscle cross-talk. AEA, arachidonoyl ethanolamide; 2-AG, 2-arachidonoyl glycerol; CB, cannabinoid receptor; COX, cyclooxygenase; cPLA_2_, cytosolic phospholipase A_2_; DAGs, diacylglycerols; DHA, docosahexaenoic acid; 10,17-DiHDoHE, 10,17-dihydroxy-docosahexaenoic acid; DP, prostaglandin D_2_ receptor; EP, prostaglandin E_2_ receptor; EPA, eicosapentaenoic acid; FLAP, 5-lipoxygenase activating protein; FP, prostaglandin F_2α_ receptor; 7-HDoHE, 7-hydroxy docosahexaenoic acid; IP, prostaglandin I_2_ receptor; LOX, lipoxygenase; LPC, lysophosphatidylcholine; LTA_4_, leukotriene A_4_; LTB_4_, leukotriene B_4_; LTC_4_, leukotriene C_4_; LTD_4_, leukotriene D_4_; LXA_4_, lipoxin A_4_; MaRs, maresins; NArPE, N-arachidonoyl phosphatidylethanolamine; OEA, oleoylethanolamide; PEA, palmitoylethanolamide; PD_1_, protectin D_1_; PGD_2_, prostaglandin D_2_; PGE_2_, prostaglandin E_2_; PGF_2α_, prostaglandin F_2α_; PGH_2_, prostaglandin H_2_; PGI_2_, prostaglandin I_2_; PPARs, proliferator-activated receptors; RvD_1_, resolvin D_1_; RvE_1_, resolvin E_1_; TG, triglycerides; TP, thromboxane receptor; TXA_2_, thromboxane A_2_

Lipids comprise a highly diverse set of molecules that play important structural and functional roles in bone and muscle. In bone, most lipids are stored in bone marrow. Changes in the composition of fatty acids in bone have been associated with reduced bone mass during aging [6, 7]. A well-documented bioactive lipid in bone is arachidonic acid (AA), which plays an essential role in bone remodeling by generation of prostaglandins via cyclooxygenases (COXs) pathways; see Fig. 1. In responding to mechanical stimulation, prostaglandin E_2_ (PGE_2_) can be released within seconds from osteocytes in vitro [8]. Mechanical loading has been shown to induce physiological plasma membrane disruptions (PMD) in osteocytes which could facilitate the quick release of lipids from osteocytes to initiate remodeling [9].

In muscle, lipids are stored inside muscle fiber (intramuscular lipid), outside muscle fiber (perimuscular and intermuscular lipids), or in plasma membrane [10, 11]. Intramuscular lipid droplets are primarily composed of triglycerides (TG) [12]. Increased intramuscular lipid deposition can result from elevated plasma fatty acid (FA) concentration and increased FA transport into skeletal muscle [13]. Under healthy conditions, intramuscular TGs can be utilized as a readily available fuel source for muscle contraction during physical exercise. However, intramuscular as well as perimuscular and intermuscular lipids can also be associated with pathological conditions, such as insulin resistance and sarcopenia [11].

For lipids in the plasma membrane, one of the critical functions is to induce intracellular signal transduction. One well-known example in skeletal muscle is phosphatidylinositol 4,5-bisphosphate (PIP2). In response to stimulations, activated phospholipase C (PLC) hydrolyzes PIP2 to form both diacylglycerol (DAG) and inositol 1,4,5-trisphosphate (IP3). IP3 will then bind to IP3 receptors to release Ca^2+^ from the endoplasmic reticulum (ER), which has important function in muscle contraction. At the same time, DAG activates protein kinase C (PKC) [14]. The PKC family consists of 15 isozymes and is involved in a range of signal transduction pathways. Siddharth et al. analyzed serum lipidomics changes of aged mice with a sarcopenic phenotype and found that levels of three lipids, lysophosphatidylinositol (LPI 16:0), phosphatidylcholine (PC 37:4), and sterol ester (SE 20:4), were elevated in aged group (18 –24 months) compared with the younger (8 months) group, whereas lysophosphatidylcholine (LPC 20:5 and LPC 20:3) levels were reduced [15]. The levels of LPC 20:5 and LPC 20:3 negatively associated with those of PC 37:4 and SE 20:4, but positively correlated with compound muscle action potential amplitude in aged group, and additionally, the level of LPC 20:3 positively correlated with gastrocnemius muscle mass, which reduced with age. Their findings suggest that disturbance in lipid metabolism may contribute to reduced muscle function and muscle mass during aging and play an important role in the development of sarcopenia [15].

### Applications of Lipidomics to Age-Related Musculoskeletal Disorders

Lipidomics is the comprehensive analysis of cellular lipids as potential biomarkers of diseases. In muscle, many lipidomics applications target TG and related bioactive lipids, especially DAG. One of the last steps in TG synthesis is esterification of diacylglycerol with fatty acids catalyzed by diacylglycerol: acyltransferase (DGAT). Lipidomics analysis of muscles obtained from dysferlin null (Bla/J) mouse, an animal model for limb-girdle muscular dystrophy 2B, reveals that distribution of TGs can be muscle-type dependent. Some TG species were specifically found in quadriceps, psoas, or gastrocnemius, but there were no unique TGs discovered in gluteus muscle. Interestingly, this muscle-specific TG distribution pattern is only displayed in the male muscles [16].

### Altered Lipid Profiles with Insulin Resistance and Aging

Since skeletal muscle is in charge of postprandial glucose disposal within the majority of body, the significant loss of skeletal muscle with aging is often accompanied by the development of insulin resistance (IR) [17]. The resulting metabolic disturbances, such as type 2 diabetes (T2D), dysregulate protein turnover and further aggravate skeletal muscle wasting [18, 19]. Currently, DAGs, ceramides, and sphingomyelins are major targeted lipids in skeletal muscle in the study of insulin resistance. In humans, muscle DAG concentration in healthy sedentary obese individuals and T2D patients were higher than that in muscle from lean endurance-trained athletes. Beyond concentration, distribution of DAG in cells determines the activation of specific isozymes of PKC. Cytosolic DAG species are more likely to activate PKCθ but not PKCε, whereas membrane DAG species prefer to activate PKCε [20]. IR induced by PKCε is through phosphorylation and inhibition of insulin receptor and cross-talk with p70 ribosomal protein S6 kinase (p70S6K) [21]. The levels of some subtypes of DAG, including C18:0/C20:4, Di-C16:0, and Di-C18:0, were significantly higher in the T2D patients, but only total membrane DAG and Di-C18:0 subtype were positively associated with IR [20]. At subcellular level of skeletal muscle in humans, sarcolemmal DAGs did not show significant correlation with insulin sensitivity, but ceramides (C18:0 species) and sphingomyelins (C18:1, C18:0, and C18:2 species) in the same compartment were inversely correlated with insulin sensitivity. In the mitochondrial, endoplasmic reticulum, and nuclear fractions, ceramides showed a similar relationship with insulin sensitivity as in the sarcolemma, but 1,2-DAGs in these fractions were positively related to insulin sensitivity [22]. The changes in mitochondrial lipids are connected with mitochondrial membrane fluidity and aging. Compared with mitochondrial membrane in young skeletal muscle, TGs increased and phosphatidylethanolamines decreased in middle age skeletal muscle, suggesting reduced mitochondrial membrane fluidity and metabolic shift [23].

Recently, Preuss et al. developed a targeted lipidomics method to quantify twelve DAG subtypes and eight ceramides in skeletal muscle and other tissues, providing a new tool for lipidomics research in skeletal muscle [24]. By using lipidomics analysis, Rivas et al. also tested the levels of separate ceramide subspecies ranging from C14:0 to C24:1 in midthigh muscle sample, obtained after an acute bout of high-intensity resistance exercise in young and older males. They found the increases of 156% and 30% in C16:0 ceramide and C20:0 ceramide separately in aging skeletal muscle, which proved the significance of ceramide in the attenuation of contractile-induced skeletal muscle adaptations [25]. Since IR and mitochondrial dysfunction are two pathological conditions associated with sarcopenia, the recent progress using lipidomics analysis in these areas will provide new direction for identification of sarcopenia biomarkers.

### Altered Lipid Profiles in Aging Bone

Compared with rapid progress in the application of lipidomics in skeletal muscle, lipidomics analysis using bone or bone marrow is rare. Recently, Zhao et al. performed lipidomics profiling in femur of ovariectomized (OVX) mouse model [26]. The identified lipid mediators consisted of TGs, fatty acyls, sphingomyelins, glycerophosphorylcholines, and sterols. Most lipids were upregulated in OVX femur, especially TGs. Only sphingomyelins and some glycerophosphorylcholines were decreased after surgery [26]. It was indicated that the disorders of these lipids led to a decrease in the differentiation and proliferation of osteoblasts and an increase with osteoclasts that induced the progression of osteoporosis. The finding in TGs is consistent with the previous data correlating TGs with osteoporosis and mirrors the changes of TGs in sarcopenia. In addition, their findings indicate that lipidomics can be well applied in the construction of the metabolic profile of bone tissue [26].

Besides bone itself, lipidomics analyses in a variety of tissues such as plasma/serum and urine can also provide hints for physical and pathological changes of bone. Association between plasma lipids and bone mineral density has also been reported in menopausal women [27]. In this study, Cabrera et al. indicated that plasma concentrations of eight glycerophospholipids (phosphatidylserine (PS) (20:4; 29:6; 31:36; 32:6; 33:6), phosphatidylethanolamine (PE) (42:1), phosphatidic acid (PA) (34:4), and phosphatidylinositol (PI) (14:0)), along with glycerolipid and sphingolipid species were significantly lower in menopausal women with low BMD while two glycerophospholipid species (phosphatidylinositol and phosphatidic acid) were elevated [27]. Another lipidomics analysis of aging bone mesenchymal stem cells (BMSCs), in which abnormal differentiation is related to the occurrence of aging osteoporosis, showed that the majority of glycerophospholipids (GPs), including phosphatidylcholines (PCs), PEs, and phosphatidylglycerols (PGs), were elevated, whereas the minority of GPs, including PAs, PIs, and PSs and sphingoid bases (SPBs) decreased in the late passage BMSCs [28]. Research conducted by Bab et al. found that some of the fatty acid amides (FAAs) (e.g., anandamide, oleoyl serine, and oleoyl ethanolamide) in skeleton stimulate osteoblast proliferation and inhibit osteoclastogenesis. These FAA results, combined with data on skeletal phenotyping of FAA receptor (CB1, CB2, GPR55)-deficient mice, show that FAAs are crucial in the regulation of skeletal remodeling [29].

The lipid mediator, PGE_2_, is the most studied bioactive lipid. PGE_2_ signaling, especially via EP4 receptor, is critical for bone mechanical properties, maintenance of bone mass, bone healing, and new bone formation [30]. PGE_2_-EP4 signaling is also important for muscle functions and muscle regeneration. Activation of EP4 receptor promotes myoblast proliferation [31], while interruption of EP4 signaling reduces muscle regeneration capacity [32]. Based on the effects of PGE_2_ on bone and muscle, targeted lipidomics analyses of PGE_2_ and its related lipid species, such as AA and other PGs, will potentially identify new biomarkers for sarcopenia and osteoporosis. It is important to note that AA interacts with other fatty acids, such as N-arachidonoylethanolamine (anandamide, AEA), oleoylethanolamine (OEA), docosahexaenoic acid (DHA), linoleic acid (LA), eicosapentaenoic acid (EPA), forming a network of bioactive lipid mediators to regulate cellular functions [33]. As shown in Fig. 1, this highlights the complex relationship between AA and a wide range of bioactive lipid mediators.

Wang et al. developed a fatty acid-targeted lipidomics method and compared fourteen lipid mediators (LMs) in young and aged gastrocnemius muscles. Among 14 targeted LMs analyzed in mice skeletal muscles, the levels of 12 different LMs, including AA and its derived eicosanoids PGE_2_, PGA_2_, and PGF_2α_, DHA, and its derivatives 8-hydroxy docosahexaenoic acid (8-HDoHE), EPA derivative 17,18-dihydroxyeicosa-5,8,11,14-tetraenoic acid (17,18-DiHETE), α-linoleic acid (n-3, ALA) metabolite 9-hydroxy-octadecatrienoic acid (9-HOTrE), LA metabolites 13-hydroxyoctadecadienoic acid (13-HODE) and 9-HODE, as well as endocannabinoid (eCB)-related mediator OEA, are significantly reduced in the aged skeletal muscles of female mice, while only 8 out of 14 LMs exhibited the age-related diminished levels in the skeletal muscles from male mice; they are AA, PGE_2_, PGF_2α_, 6-keto-PGF_1α_, 8-HDoHE, OEA, and another eCB-related mediator AEA. This age-related reduction is observed to be more prominent in females than in males. Additionally, opposing altered DHA levels between male and female during aging is possibly due to hormonal influences [34].

More comprehensive lipidomics profiling methods have targeted lipid metabolites in skeletal muscle studies [35]. During myogenic differentiation of mouse primary myoblasts, knocking down COX-1 and -2 significantly inhibits myogenesis and compromises intracellular calcium signaling. These inhibitory effects are related to reduced levels of PGE_2_ and 15-hydroxyeicosatetraenoic acid (15-HETE), a lipid derived from AA through lipoxygenase (LOX) pathway. These data demonstrate that COXs and LOXs could interact with each other to regulate muscle mass and functions. Moreover, release of OEA from muscle cells also was reduced after knocking down COXs, which decreases osteoclast function and inhibits survival of mature osteoclasts through GPR119-dependent or -independent pathways [35].

## Conclusion

Bone and muscle tissues participate in a wide range of systemic metabolic functions through the secretory osteokines and myokines. Myokines could impact bone formation through regulation of osteogenesis, insulin resistance, and inflammation, while bone-derived osteokines like osteoprotegerin (OPG) can protect skeletal muscle from atrophy [36, 37]. An increasing body of research is showing that bioactive lipids are a critical subset of osteokines and myokines. These signaling molecules, generated from muscles or bones in response to extracellular stimuli, can interact with specific receptors or affect the production of other osteokines/myokines to regulate the functions of bone, muscle, and other organs/tissues through autocrine, paracrine, and endocrine pathways (Fig. 1) [37]. Additionally, these bioactive lipids can also affect the production of other osteokines/myokines. Arachidonic acid-derived lipid mediator prostaglandin D_2_ (PGD_2_) in human primary osteoblasts can significantly reduce the production of two osteokines—OPG by binding with PGD_2_ receptor (DP), and receptor activator of nuclear factor-kappaB ligand (RANKL) by activating chemoattractant receptor-homologous molecule expressed on TH2 cells (CRTH2), respectively [38]. Therefore, PGD_2_ could induce bone resorption or bone formation depending on which receptor activation occurs. The reciprocal regulation of PGE_2_ and interleukin 6 (IL-6), an important myokine-regulating osteoblast/osteoclast activities and exercise capacity, also suggests that bioactive lipids could be important for maintaining bone and muscle function during aging through regulating myokine/osteokine signaling [39–41].

As described in this review, recent studies, taking advantage of new lipidomics methods are making significant advances in the identification of potential biomarkers for the detection and diagnosis of sarcopenia and osteoporosis. These studies are also leading to new mechanistic insights into the changes in metabolic perturbations that accompany the development of age-related MSDs. Continued progress in this area could lead not only to improved detection, but the identification of new therapeutic targets to combat these devastating diseases.
